# Strong Coupling of Carbon Quantum Dots in Liquid Crystals

**DOI:** 10.1021/acs.jpclett.1c03937

**Published:** 2022-04-15

**Authors:** Sema Sarisozen, Nahit Polat, Fadime Mert Balci, C. Meric Guvenc, Coskun Kocabas, Halime Gul Yaglioglu, Sinan Balci

**Affiliations:** † Department of Chemistry, Izmir Institute of Technology, Izmir 35430, Turkey; ‡ Department of Photonics, Izmir Institute of Technology, Izmir 35430, Turkey; § Department of Materials Science and Engineering, Izmir Institute of Technology, Izmir 35430, Turkey; ∥ Department of Materials, University of Manchester, Manchester M13 9PL, U.K.; ⊥ National Graphene Institute (NGI), University of Manchester, Manchester M13 9PL, U.K.; # Henry Royce Institute for Advanced Materials, University of Manchester, Manchester M13 9PL, U.K.; ∇ Department of Engineering Physics, Ankara University, Ankara 06100, Turkey

## Abstract

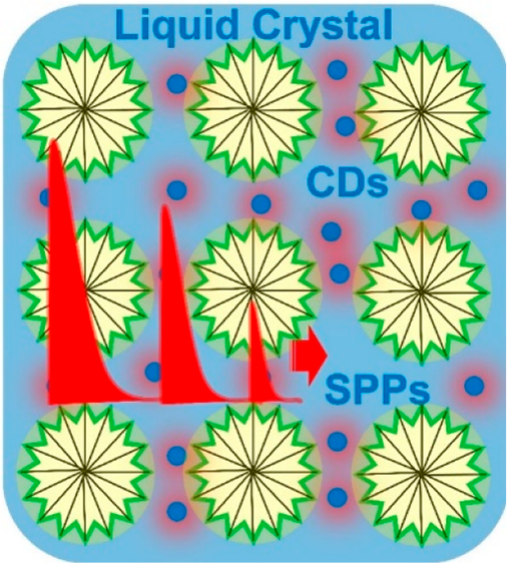

Carbon quantum dots (CDs) have recently received a tremendous amount of interest owing to their attractive optical properties. However, CDs have broad absorption and emission spectra limiting their application ranges. We herein, for the first time, show synthesis of water-soluble red emissive CDs with a very narrow line width (∼75 meV) spectral absorbance and hence demonstrate strong coupling of CDs and plasmon polaritons in liquid crystalline mesophases. The excited state dynamics of CDs has been studied by ultrafast transient absorption spectroscopy, and CDs display very stable and strong photoluminescence emission with a quantum yield of 35.4% and a lifetime of ∼2 ns. More importantly, we compare *J*-aggregate dyes with CDs in terms of their absorption line width, photostability, and ability to do strong coupling, and we conclude that highly fluorescent CDs have a bright future in the mixed light–matter states for emerging applications in future quantum technologies.

Carbon based nanomaterials including graphene, carbon nanotubes, nitrogen vacancy centers in nanodiamond, carbon nanofibers, fullerene, and recently carbon quantum dots have found a wide range of applications in basic and applied science owing to their superior physical and chemical properties. Luminescent carbon nanostructures such as semiconducting carbon nanotubes^1^ and carbon dots (CDs), also known as carbon quantum dots or graphene quantum dots,^2^ are very attractive, since they have huge potential in many applications, from optoelectronic devices to bioimaging.^3,4^ Among the carbon nanomaterials, CDs, a zero-dimensional carbon nanomaterial, serendipitously discovered during the purification of carbon nanotubes in 2004,^2^ are fluorescent semiconductor nanocrystals with tunable optical and optoelectronic properties in the visible and near-infrared region of the electromagnetic spectrum. Both top-down and bottom-up approaches have been widely employed in order to synthesize carbon quantum dots. For instance, laser ablation of graphite powder,^5^ separation of impurities (carbon quantum dots) from arc-synthesized single-walled carbon nanotubes,^2^ electrochemical conversion of carbon nanotubes into CDs,^6^ and hydrothermal, solvothermal, and microwave synthesis of CDs from organic precursors^7,8^ are some of the synthesis techniques used for CDs. In fact, the bottom-up approach is more attractive, since conjugated and non-conjugated carbon precursors are easily and effectively converted to CDs, and the chemical and optical properties of the resulting nanocrystals can be easily modified during the synthesis. However, when compared with cadmium and lead based semiconducting quantum dots and fluorescent dyes, CDs have unique advantages, such as bright luminescence, heavy-metal-free, low toxicity, low cost, biocompatibility, facile and easy synthesis, water solubility, and photostability, and therefore, they have attracted a great amount of interest in optoelectronic devices,^9^ bioimaging, drug delivery,^10^ and photodynamic therapy^11^ as well. However, the synthesized CDs usually have broad absorption and emission peaks, which are tunable in the visible region.^12^ In a recent study, narrow band emitting triangular carbon quantum dots have been demonstrated.^13^ Although the triangular CDs, synthesized by using the solvothermal method at 200 °C and purified by using column chromatography, have very narrow bandwidth emission (around 30 nm) with a quantum yield of more than 50%, the reaction yield is very low. Therefore, in order to synthesize narrow bandwidth red emitting CDs and hence expand the application of red emitting CDs in a variety of fields such as light emitting diodes, and strong light–matter interactions, new synthetic methods for the formation of CDs with large reaction yields and superior optical properties remain a key goal and challenge.

In the strong coupling regime, the excitons of semiconducting quantum dots or dyes interact strongly with surface plasmon polaritons (SPPs) and new hybrid plasmon–exciton states, i.e., plexcitons, emerge.^14,15^ In a recent study, a single colloidal quantum dot of CdSeTe/ZnS has been used to show deterministic room temperature strong coupling of a colloidal quantum dot to a plasmonic nanocavity at the apex of a scanning probe.^16^ At the ensemble level, the dynamics of strong coupling between the excitons of CdSe quantum dots and surface plasmon polaritons of the gold nanohole array has been investigated.^17^ In another recent study, a plasmonic cavity containing a few semiconducting quantum dots has shown complex plasmon–exciton dynamics revealed through quantum dot light emission in a nanocavity.^18^ Until now, various metal thin films and Ag/Au nanostructures such as nanorods, nanoprisms, nanodisks, nanorings, and excitonic sources such as colloidal quantum dots, organic dyes, *J*-aggregates, and two-dimensional nanomaterials have been widely employed in order to observe a plasmon–exciton mixed state in the strong coupling regime.^19−21^ However, up until now, colloidal CDs have not been employed for the observation of plasmon–exciton mixed states. In this study, we, for the first time, report high-yield hydrothermal synthesis of water-soluble red emissive CDs displaying a very narrow excitonic line width and place them in lyotropic liquid crystalline mesophases for the observation of a plasmon–exciton mixed state near a thin metal film. The CDs synthesized on a relatively large scale through a facile and simple method and not purified by using column chromatography show high reaction yield and very stable and strong photoluminescence (red emission) with a quantum yield of around 35.4% and a lifetime of around 2 ns. In addition, the excited state dynamics of CDs has been extensively studied by using ultrafast transient absorption spectroscopy. In order to study the interaction of excitons of CDs and surface plasmon polaritons (SPPs) of thin metal film, CDs have been dispersed in the synthesized lyotropic liquid crystalline (LLC) mesophases and hence a red emitting LLC mesophase–CD film has been fabricated and placed near a metal film. Polarization dependent spectroscopic reflection measurements show that the excitons of CDs and SPPs of thin metal film interact strongly and thus a plasmon–exciton hybrid state has been observed. More importantly, we compare CDs with *J*-aggregate dyes in terms of their absorption line width, photostability, and ability to do strong coupling, and therefore, we conclude that highly fluorescent carbon quantum dots with very narrow absorption line widths, high quantum yields, high photostability, low toxicity, low cost, and tunable emission in the visible and near-infrared region of the electromagnetic spectrum have a bright future in light–matter interaction studies in the nanoscale dimension. Apart from the previous studies, the present study shows the following: (i) strong coupling between excitons of CDs and SPPs of thin metal film in the liquid crystals, (ii) high yield (not purified by column chromatography) hydrothermal synthesis of CDs with very narrow absorption and emission peaks, (iii) dispersion of carbon quantum dots in lyotropic liquid crystalline mesophases, (iv) excited state dynamics of the synthesized CDs, and (v) comparison of the optical properties of *J*-aggregates (commonly used dye in the strong light–matter interaction studies) with CDs.

## Experimental Section

### Chemicals

l-Phenylalanine, *o*-phenylenediamine (OPD), sodium hydroxide (NaOH), and sulfuric acid (95%) were all purchased from Sigma-Aldrich and used without any further purification. 5,5′,6,6′-Tetrachlorodi(4-sulfobutyl)benzimidazolocarbocyanine (TDBC) dye showing *J*-aggregate properties at high concentration in aqueous medium was purchased from FEW Chemicals GmbH. Milli-Q water with a resistivity of 18.2 MΩ·cm was used in all of the experiments.

### Synthesis of Carbon Dots (CDs)

The carbon quantum dots were synthesized by using a hydrothermal method at high temperature in a Teflon-lined stainless steel autoclave. In a typical procedure, 0.0649 g (0.6 mmol) of *o*-phenylenediamine and 0.0991 g (0.6 mmol) of l-phenylalanine were dissolved in 8 mL of ultrapure water and placed in a 25 mL Teflon-lined stainless steel autoclave. The mixture was stirred at room temperature in order to completely dissolve all chemicals in the mixture. In addition, 2 mL of sulfuric acid (95%) was added into the solution in order to increase the solubility of the precursors and catalyze the reaction. When *o*-phenylenediamine and l-phenylalanine were completely dissolved in a 8:2 mixture of ultrapure water and sulfuric acid, the resulting solution appeared as a transparent solution. After sufficient dissolution of the precursors, the Teflon-lined stainless steel autoclave was placed in a preheated oven and then the oven temperature was set to 210 °C for 12 h. Subsequently, after the reaction was complete within 12 h, the Teflon-lined stainless steel autoclave was cooled down naturally to room temperature. The resulting solution was dark-blue-colored. In order to remove large aggregates in the reaction solution, the dark blue solution was centrifuged at 15,000 rpm for 15 min. After centrifugation, the pellet was discarded and the supernatant was filtered through a 0.22 μm filter. The centrifuged and filtered solution was kept at 4 °C for storage. It is noteworthy that CD colloids are also very stable at room temperature. The obtained CD colloid in water exhibits strong red fluorescence with a quantum yield (QY) of 23.4% in water. The CDs in ethanol show a quantum yield of 35.4%. The final concentration of the red luminescent carbon quantum dots is around 0.08 mg/mL. In order to precipitate the CDs, the solution was neutralized with a dropwise addition of NaOH under continuous stirring. After neutralization, the precipitate was washed and centrifuged three times at 15,000 rpm for 15 min in order to remove excess NaOH. Finally, the pellet was redispersed in a variety of buffer solutions for photoluminescence measurements. It was also observed that the fluorescence of the CDs is strongly pH dependent.

### Preparation of Lyotropic Liquid Crystalline (LLC) Mesophases

The LLC mesophase of sulfuric acid (SA) (a strong acid as a solvent) and 10-lauryl ether (C_12_E_10_) (a non-ionic surfactant) was prepared by using a SA/C_12_E_10_ mole ratio of 2.5, which forms a very stable hexagonal mesophase and displays a typical fan texture under the polarized optical microscope. In order to obtain the lyotropic liquid crystalline film, first, the mesophase was prepared as a solution in ethanol. Subsequently, the solution was coated over a substrate via a spin coater, and hence, the gel phase was formed as a thin film.

### Characterization

In order to investigate the morphology of the nanocrystals, a scanning transmission electron microscope (STEM) (SEM; Quanta 250, FEI, Hillsboro, OR, USA) and high resolution transmission electron microscope (HRTEM) (FEI 120 kV Jeol HRTEM) were used. The samples were prepared by drop-casting CD suspensions onto 200 mesh formvar/carbon coated copper grids. The extinction measurements of the CDs in a 1 cm quartz cuvette were performed by using a balanced deuterium–tungsten halogen light source (DH2000-BAL, Ocean Optics) and a fiber coupled spectrometer (USB4000, Ocean Optics). All characterization measurements were carried out under ambient conditions. A polarizing optical microscope was used to characterize LLC thin films. In the optical path of the illumination, the polarizing microscope has a polarizing filter (a polarizer) below the sample stage and a polarizing filter (an analyzer) above the sample stage. The polarizing optical microscope images were obtained in the transmission mode. Photoluminescence (PL) and time-resolved lifetime (LT) measurements of CDs were carried out by using a FS5 Spectrofluorometer (Edinburgh Instruments, UK). During the PLQY measurements, a xenon lamp was employed with an excitation wavelength of 350 nm. An integrating sphere was used in PLQY of each sample. The QY measurements were performed in a cylindrical quartz cuvette. During the LT measurements, the CD colloids were excited with a 350 nm laser having a pulse width of 100 ps and a repetition rate of 1 MHz. Ultrafast transient absorption spectroscopy (fs-TA) experiments were carried out with a 610 nm pump wavelength, about 40 fs pulse duration, and 1 kHz repetition rate. The change of the absorption intensity (Δ*A* = Δ*T*/*T*) of the sample after 610 nm pump excitation is plotted as a function of the probe wavelength for various time delays. In the global fitting, the transient absorption data were analyzed by using a software program called Surface Xplorer PRO from Ultrafast Systems. The software allows us to perform temporal chirp corrections, time zero adjustment, and deconvolution from the instrumental response function. In fact, global fitting revealed carrier lifetimes as 0.41 + 0.01 ps, 0.13 + 0.07 ns, 1.5 + 0.5 ns, and decay time, which is longer than our maximum delay (>3.3 ns).

### Numerical Calculations

The finite difference time domain (FDTD) method was employed to investigate the optical properties of CDs near flat Ag films. In the simulations, a plane wave was used. Polariton dispersion curves of the flat and bare silver films representing the uncoupled surface plasmon polaritons were obtained. The SPPs of the flat metal film at different resonance frequencies were excited by varying the incidence angle of the incident light. For example, when the incidence angle is 45° for a bare silver film, the SPP resonance wavelength is around 600 nm. The mesh size was 5 nm during the polariton dispersion curve calculations. The FDTD simulation of plasmon–exciton coupling was investigated in the Kretschmann configuration. A prism was used to couple incident light to surface plasmons of metal film. The excitons of the CDs were assumed to be a Lorentzian line shape and expressed as ε(ω) = ε_*∞*_ + *f*_0_(*ω*_0_^2^/(*ω*_0_^2^ – ω^2^ – *i*γ_0_ω)) where the resonance wavelength of the oscillator was set to 625 nm (1.984 eV) and the width of the resonance (γ_0_) was set to around 95 meV. The background index, *ε*_*∞*_, was set to 2.1. The calculated dispersion curves of the bare and coupled metal films with collection of CDs in the liquid crystal were obtained by acquiring the reflection spectra for each incidence angle within a broad wavelength range, and then the resulting reflectivity distribution for each incidence angle was obtained in heat maps, which clearly exhibit the degree of coupling between SPPs of flat silver film and excitons of CDs in the liquid crystal.

### Plasmon–Exciton Coupling

In order to study plasmon–exciton coupling of CDs embedded in the LLC mesopahses on flat metal surfaces, a well-known Kretschmann configuration was used. The surface plasmons on thin (∼40 nm) metal films were excited with an incident light.^22^ In the strong coupling regime, incident photons interact strongly with the surface plasmons on the metal film, and thus, surface plasmon polaritons are generated in the strong coupling regime. Ag films with thicknesses of 25, 50, and 60 nm on glass substrates were grown by thermal evaporation of Ag under a vacuum. A piranha solution, a 3:1 mixture of sulfuric acid (95%) with hydrogen peroxide (30%), was used to remove organic residues from the glass substrates before silver evaporation. The polariton dispersion curves of coupled and uncoupled silver films were generated by using a tunable laser with a spectral width of around 1 nm, i.e., a supercontinuum laser (Koheras-SuperK Versa) with an acousto-optic tunable filter working in the visible region of the electromagnetic spectrum. A glass prism made of BK7 was used to excite surface plasmons on the Ag films.

**Figure 1:**
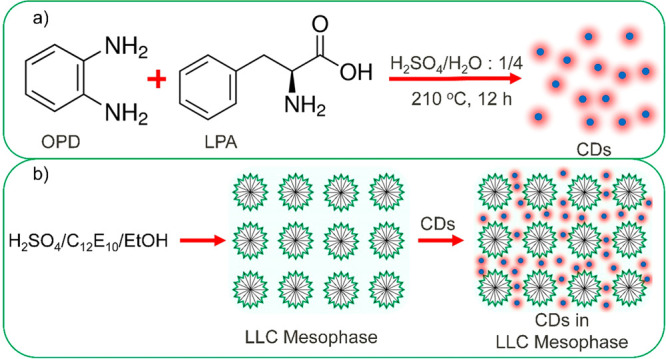
Synthesis of carbon quantum dots and lyotropic liquid crystalline mesophases. (a) Hydrothermal synthesis of CDs from ortho-phenylenediamine (OPD) and l-phenylalanine (LPA) in a water–sulfuric acid mixture at a high temperature, 210 °C. The optimum sulfuric acid to water ratio is found to be 0.25. (b) Preperation of lyotropic liquid crystalline (LLC) mesophases from the sulfuric acid, surfactant, C_12_E_10_, and ethanol. The synthesized CDs are dispersed in LLC mesophases. The CDs in LLC mesophases emit red light.

The CDs were synthesized via a simple hydrothermal strategy from ortho-phenylendiamine (OPD) and l-phenylalanine (LPA) precursors in the water–sulfuric acid mixture at high temperature in a Teflon-lined stainless steel autoclave for 12 h, Figure 1a. After the reaction was complete, the Teflon-lined stainless steel autoclave was cooled down to room temperature naturally. In fact, the resulting solution was dark-blue-colored. During the hydrothermal reaction, the solution changes its color from transparent to dark-blue, which is a very strong indication of the CD formation. Large aggregates formed during the hydrothermal reaction were removed by centrifugation. The final concentration of the red luminescent carbon quantum dots is around 0.08 mg/mL. In order to precipitate the CDs, the solution was neutralized with a dropwise addition of NaOH under continuous stirring conditions. After neutralization, the precipitate was washed and centrifuged three times in order to remove excess NaOH. Finally, the pellet was redispersed in a variety of buffer solutions for photoluminescence measurements. It was also observed that the fluorescence of the CDs is strongly pH dependent; see the Supporting Information for the pH dependent 2D excitation–emission topographical maps of CDs.

It is noteworthy that CD colloids are very stable at room temperature. The optimum sulfuric acid to water ratio has been found to be around 0.25. The synthesized CDs were dispersed in LLC mesophases, Figure 1b. In fact, the amphiphilic molecules form self-assemblies in a variety of media such as water, organic solvents, ionic liquids, salts, and acids. Non-ionic surfactants can form lyotropic liquid crystalline mesophases in hygroscopic species such as salts and acids. The non-ionic surfactant, 10-lauryl ether (C_12_E_10_), forms a very stable lyotropic liquid crystalline mesophase by using a strong acid, sulfuric acid as a solvent between 2 and 12 sulfuric acid/C_12_E_10_ mole ratio.^23^ The water uptake of the system increases with the amount of sulfuric acid. The typical water uptake of the stable C_12_E_10_/sulfuric acid lyotropic liquid crystal increases from 2.3 to 4.3 per sulfuric acid when the amount of sulfuric acid increases from 2 to 12 per C_12_E_10_. The mixture of 10-lauryl ether and sulfuric acid with a little amount of water forms a two-dimensional hexagonal mesophase up to around 3.5 sulfuric acid per C_12_E_10_ and transforms to a micelle cubic phase above this ratio. While the hexagonal mesophase displays a typical fan texture under a polarizing optical microscope, a cubic phase shows no texture formation, since the cubic phase is optically isotropic and hence appears black under a polarizing optical microscope. In this study, we worked with a 2.5 sulfuric acid/C_12_E_10_ mole ratio, which forms a very stable hexagonal mesophase and displays a typical fan texture under a polarizing optical microscope; see the Supporting Information for the polarizing optical microscope images of LLC mesophases and LLC mesophases containing CDs. The LLC mesophases were prepared from sulfuric acid, surfactant, C_12_E_10_, and ethanol. Owing to the presence of sulfuric acid in the LLC mesophases, the CDs emit red light. The red emitting LLC mesophases with embedded CDs enable red emitting thin film formation of CDs. In order to obtain the LLC film, first, the mesophase is prepared as a solution in ethanol. After coating the solution over a substrate via a spin coater, the gel phase is formed as a thin film. It should be emphasized here that we were not able to prepare stable and uniform CD thin films by using poly(vinyl alcohol) (PVA), which might be due to the degredation of the polymer film in the strongly acidic medium.^24^

**Figure 2:**
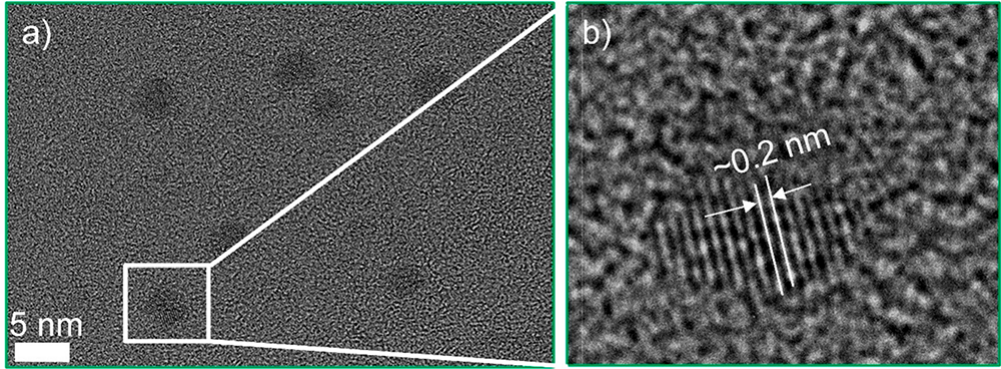
High-resolution TEM micrographs depicting the atomic resolution of CDs. (a) Large area TEM micrograph of CDs showing an average diameter of less than 5 nm. (b) A magnified HRTEM image of a single carbon quantum dot on a carbon coated copper grid. A *d* spacing of around 0.2 nm can be calculated from the zoomed-in HRTEM image of the highligted region in part a.

The HRTEM image of the CDs is shown in Figure 2a. The zoomed-in HRTEM image of the selected area marked by the white rectangle in Figure 2a is shown in Figure 2b. Indeed, CDs shown in Figure 2a possess a crystalline structure. The CDs have well-resolved lattice fringes with a lattice spacing of around 0.2 nm, which can be attributed to the in-plane lattice spacing of the graphene (100) facet.^25^ In fact, HRTEM images of the nanocrystals demonstrate defects in their crystalline structure. The average size of the nanocrystals is around 4 nm. Furtheremore, the FTIR spectrum of the as-synthesized CDs is shown in Figure S4, which displays critical information about the functional groups in the CDs. The CDs have indeed the stretching vibration of C—OH (3300 cm^–1^), the stretching vibration of C—H (2920 cm^–1^), vibrational absorption of C=O (1730 cm^–1^), and C=C (1620 cm^–1^) peaks in the spectrum.^26^

**Figure 3:**
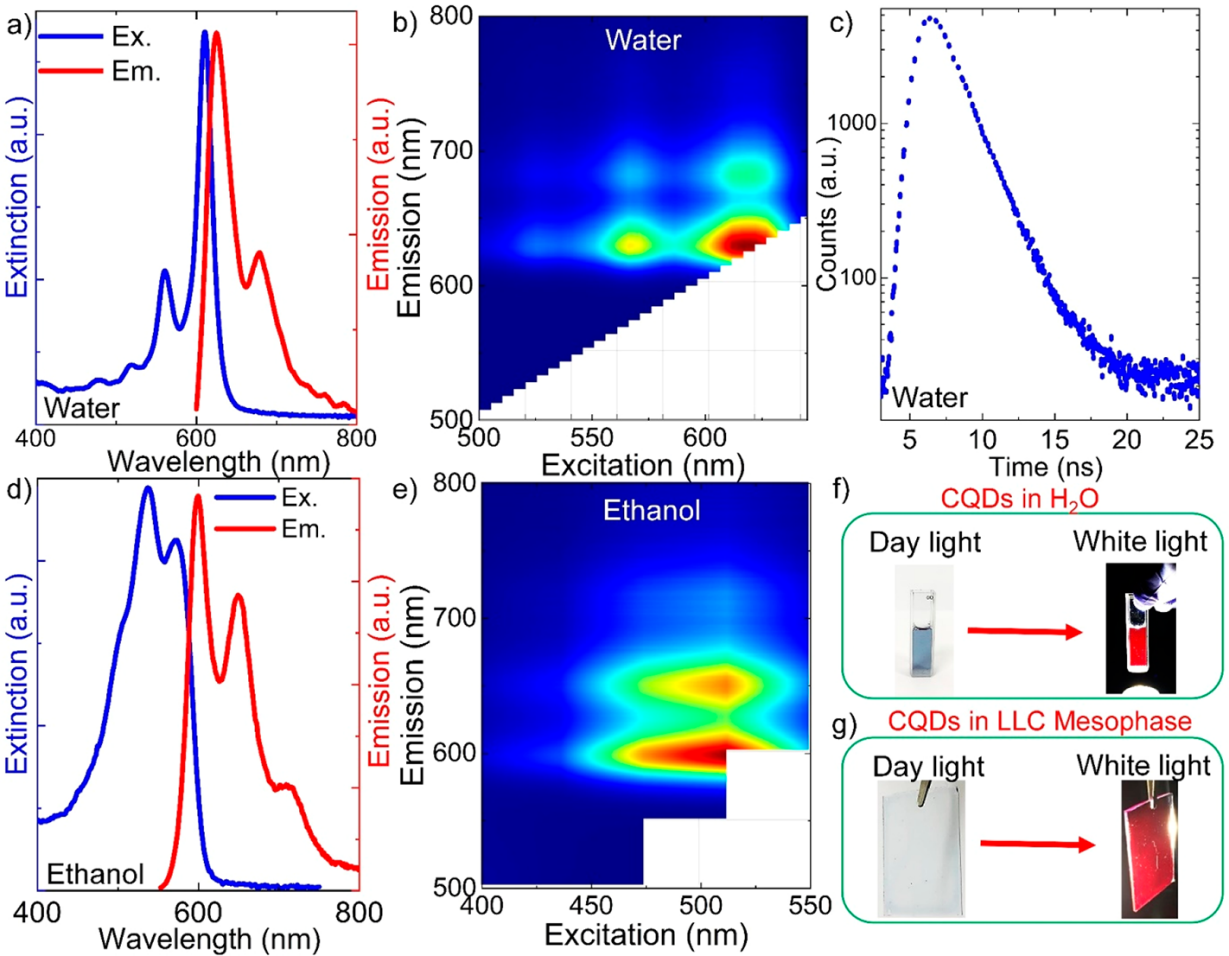
Linear optical properties of CDs. (a) Extinction and photoluminescence (PL) spectra of CDs in water. The fwhm of the absorption peak is around 75 meV (∼22 nm). (b) 2D excitation–emission topographical map of CDs in water. The red-colored region indicates the maximum PL emission intensity, whereas the blue-colored region in the map shows the minimum PL emission intensity. (c) PL lifetime of CDs in water. The estimated lifetime of CDs is around 2 ns. (d) Extinction and PL spectra of CDs in ethanol. (e) 2D excitation–emission topographical map of CDs in ethanol. (f) Colloidal CDs placed in a quartz cuvette seen under daylight illimunation and intense white light excitation. (g) CDs dispersed in the lyotropic liquid crystalline mesophase and then spin coated on a glass surface seen in daylight illumination and intense white light excitation. CDs emit red color both in water and in the thin film of the LLC mesophase.

After the synthesized CDs were isolated and redispersed in the acidic solution, the CDs exhibited red photoluminescence under visible light irradiation, as shown in Figure 3. The CDs show a very narrow and strong absorption peak at around 610 nm and a strong emission peak at around 624 nm in the spectra, Figure 3a. The UV–vis absorption spectra of the CDs were measured by using a UV–vis spectrophotometer. In the UV region, the absorbance bands at around 280 nm are due to the π–π* transitions of C=C and C=N double bonds. The fluorescence emissions of the CDs in water and ethanol are due to the absorption of the CDs in the lower region of the absorption spectrum (500–650 nm). Also, the CDs have very weak absorption peaks at around 560, 517, and 477 nm. The full width at half-maximum (fwhm) of the strong absorption and emission peaks are around 22 nm (around 75 meV) and 35 nm (around 120 meV), respectively. In a previous study, CDs synthesized from taxus leaves by the solvothermal method and then purified via silica gel column chromatography showed a very narrow fwhm of around 20 nm.^27^ In another study, triangular CDs syhthesized from organic compounds demonstrated narrow excitonic emission peaks with very narrow fwhm values of around 30 nm.^13^ The optical properties of CDs were extensively studied in acidic aqueous solutions at room temperature. The excitation–emission color map of the CDs was shown in Figure 3b, which indicates that the spectral position of the strong emission peak at around 624 nm and a weak emission peak at around 680 nm remained in their positions for all of the excitation wavelengths. Obviously, all of the emission spectra of the CDs are strictly excitation wavelength independent. In order to measure the quantum yield (QY) of CDs, we used time-resolved single photon counting measurement. The QYs of the CDs in water and ethanol were measured and are shown in the Supporting Information (Figure S8). In fact, the CDs have high QYs both in water and in ethanol. The synthesized colloidal CDs exhibit the highest QY of 35.4% in ethanol for red fluorescence emission. However, the CDs exhibit a strong red fluorescence with a lower QY of 23.4% in water. In addition, an estimated lifetime of around 2 ns was measured in water; see Figure 3c for the PL lifetime of CDs in water. The low QY of CDs in water may be due to the poor solubility and therefore the aggregation of the CDs in water. Consequently, the fluorescence emission of the CDs may be quenched, owing to the excessive energy transfer or direct π–π interactions between the CDs.^28^Figure 3d shows the absorption and emission spectra of CDs in ethanol. The excitation–emission color map of the CDs in ethanol is shown in Figure 3e, which indicates that the spectral positions of the emission peaks remain in their positions for all of the excitation wavelengths. There is also a very small and broad shoulder peak at around 713 nm. The excitation–emission map difference in water and ethanol implies that the CDs have different energy gaps in different solvents.^29^ In a previous study, it was observed that, as the solvent polarity increased, the absorption wavelength showed a red shift.^29^ Besides, colloidal CDs in water placed in a quartz cuvette seen in daylight illimunation (blue-colored) and under intense white light excitation (red-colored) are shown in Figure 3f, respectively. Furtheremore, the CDs were dispersed in the lyotropic liquid crystalline phase and then they were spin coated on a glass substrate, as seen under the daylight illimunation and intense white light excitation in Figure 3g. Importantly, the CDs emit strong red light both in aqueous solution and in LLC mesophase thin film as well.

**Figure 4:**
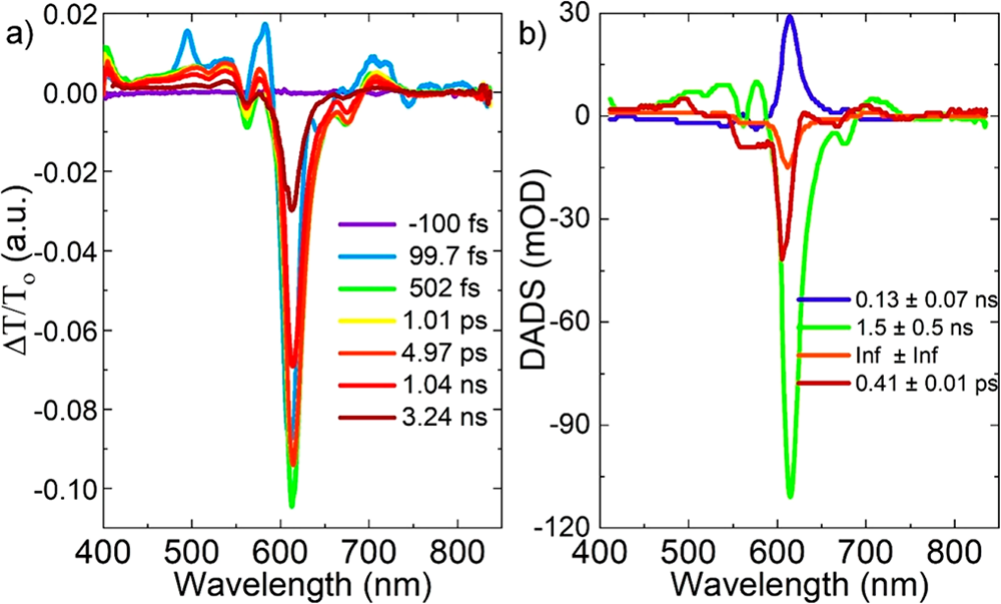
Transient absorption spectra of CDs in water. (a) The variation of the absorption intensity (Δ*A* = Δ*T*/*T*) of the sample after 610 nm pump excitation is plotted as a function of probe wavelength for various time delays. (b) Spectral evolution occurring within the carrier lifetime constants of CDs given as decay associated differential spectra (DADS).

The excited state dynamics of the colloidal CDs in aqueous medium were investigated by means of ultrafast transient absorption spectroscopy. The ultrafast transient absorption spectroscopy (fs-TA) experiment was carried out with a 610 nm pump wavelength, about 40 fs pulse duration, and 1 kHz repetition rate. The change of the absorption intensity (Δ*A* = Δ*T*/*T*) of the sample after 610 nm pump excitation is plotted as a function the probe wavelength for various time delays, as shown in Figure 4a. The negative absorption signals at around 610 nm represent ground state bleaching (GSB) and stimulated emission (SE). However, the weaker positive absorption signals seen below and above the negative absorption peaks indicate excited state absorption (ESA). Besides the strong bleaching signal at 610 nm pump wavelength, there are weak bleaching signals above and below the pump wavelengths as well. In an attempt to reveal various relaxation channels of the excited carriers in CDs, global analysis was performed on fs-TA. Global fitting revealed carrier lifetimes as 0.41 + 0.01 ps, 0.13 + 0.07 ns, 1.5 + 0.5 ns, and decay time, which is longer than our maximum delay (>3.3 ns). Spectral evolution happening within these time constants is given as decay associated differential spectra (DADS) in Figure 4b. These results are similar to DADS obtained in a recent work about NBE-T-CDs.^13^ It is known that hot electrons generated upon excitation thermalize within a few tens of a femtosecond, which is less than our pulse duration (about 50 fs) and, therefore, beyond our time resolution.^30^ In fact, the 0.41 ± 0.01 ps time component is very close to the 0.54 ± 0.01 ps component obtained for NBE-T-CDs,^13^ which was attributed to energy transfer from the thermalized carriers to the optical phonons.^31^ In addition, the 0.13 ± 0.07 ns time component can be attributed to non-radiative transition into the ground state. More importantly, the 1.5 ± 0.5 ns time component corresponds to the radiative transition into the ground state, which is very close to the experimentally measured fluorescence lifetime of around 2 ns. The radiative decay component within 1.5 ± 0.5 ns has a much higher amplitude than that of the non-radiative decay component within the 0.13 ± 0.07 ns time window, as shown in Figure 4b. In other words, the non-radiative excited state relaxation process, which is responsible for broadening of the emission peak, has a smaller contribution than that of the radiative excited state relaxation. This is indeed the explanation of the high-color-purity excitonic emission obtained from our samples.

**Figure 5:**
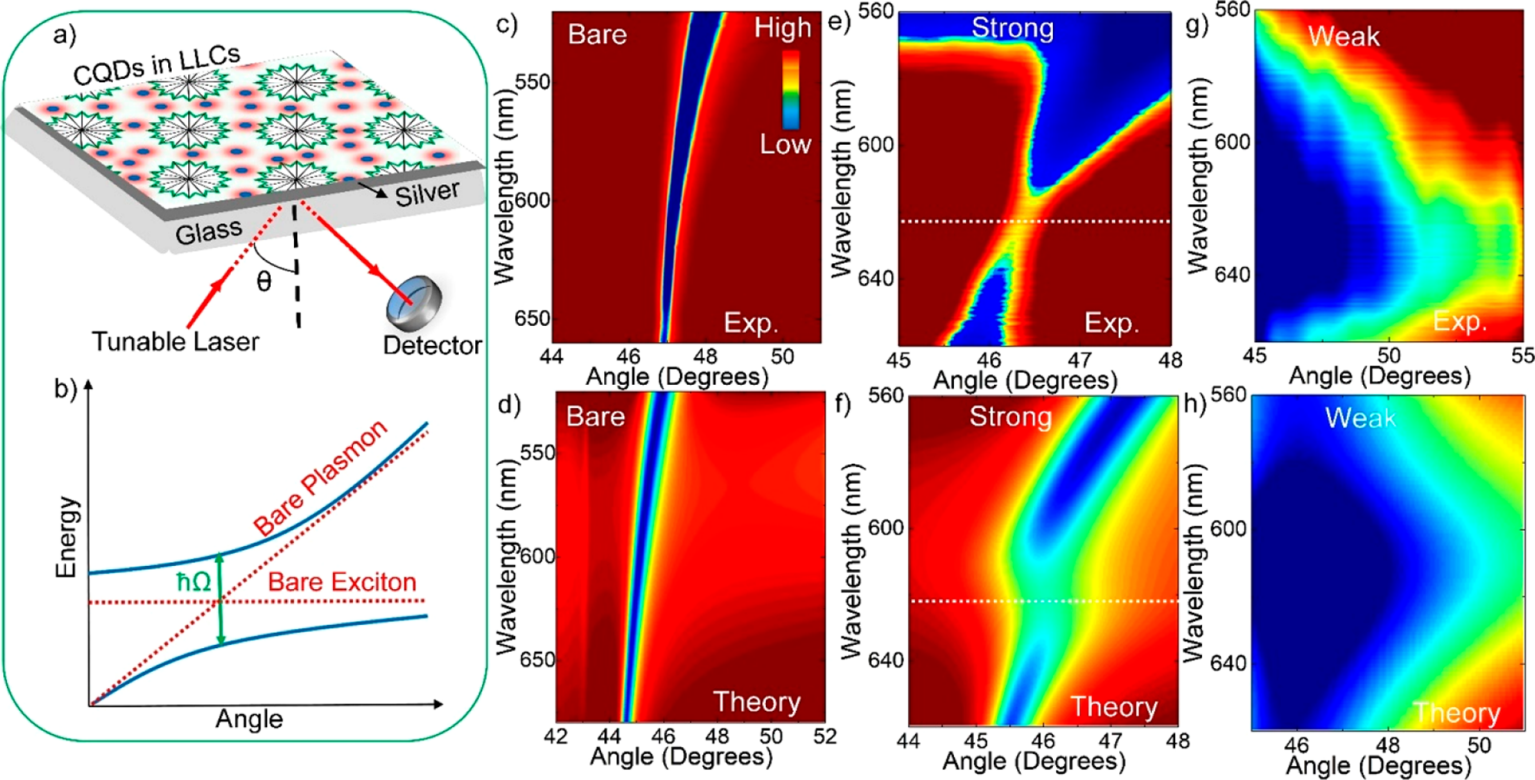
Strong and weak light–matter coupling in carbon quantum dots embedded in LLC mesophases placed near a metal thin film. (a) Schematic diagram of the experimental setup, Kretschmann configuration, used for the excitation of surface plasmons on the flat silver film covered with CDs in LLC mesophases. (b) Schematic representation of the strong coupling observed between excitons of CDs and surface plasmon polaritons of metal film. In the strong coupling regime, an anticrossing is observed. The Rabi splitting energy calculated from the polariton dispersion curve represents the degree of coupling. (c) Experimentally obtained surface plasmon polariton dispersion curve from a bare silver film. (d) Theoretically obtained surface plasmon polariton dispersion curve from a bare silver film. (e) Experimentally obtained dispersion curve from a 60 nm thick silver film covered with CDs. (f) Theoretically obtained polariton dispersion curve of silver film covered with a Lorentz oscillator having the same resonance wavelength and line width as the CDs. The red and blue colors in the polariton dispersion curves represent high and low reflectivity, respectively. At around 625 nm (resonance wavelength of the CDs in the liquid crystal), an anticrossing was observed in the experimental and theoretical dispersion curves. (g) Experimentally obtained dispersion curve from a 25 nm thick silver film covered with CDs. (h) Theoretically obtained polariton dispersion curve of a 25 nm thick silver film covered with a Lorentz oscillator having the same resonance wavelength and line width as the CDs.

We now turn our attention to strong and weak light–matter interaction in collection of CDs embedded in the LLC mesophase placed near a metal thin film. In order to study plasmon–exciton coupling^15^ in CDs on flat metal surfaces, a well-known Kretschmann configuration, as shown in Figure 5a, was used. It should be noted that the Kretschmann configuration is commonly used for the excitation of surface plasmons on thin metal films.

In fact, incident photons interact with the surface plasmons on the surface of thin metal films and hence surface plasmon polaritons (SPPs) are generated. When the horizontal component of the incident light momentum (*k*_*x*_) is equal to the real part momentum of surface plasmons (*k*_SP_), the SPPs are formed. SPP resonance occurs indeed when the incident light frequency is equal to the surface plasmon frequency. The dispersion relation is *k*_*x*_ = *k*_o_*n*_p_ sin(θ) = *k*_SP_ = 2π/λ(*ε*_m_*ε*_d_/ε_m_ + ε_d_)^1/2^, where *n*_p_ is the refractive index of a prism, θ is the angle of incident light, λ is the wavelength of incident light, and *ε*_m_ and *ε*_d_ are the dielectric constants of metal and dielectric, respectively. Polarized reflection measurements were performed for each incidence angle to study coupling between excitons of CDs and SPPs of metal film, Figure 5b. Figure 5c shows the dispersion curve from a thin Ag film obtained by using a tunable laser light source with a spectral width of around 1 nm. Additionally, to corroborate the experimental results obtained, theoretical calculations were performed, Figure 5d. When the Ag film was covered with CDs in LLC mesophases, an anticrossing behavior in the dispersion curve was observed. It should also be noted here that CDs in water and in LLC mesophases have maximum absorbance resonance wavelengths of around 610 and 625 nm, respectively (see Supporting Information, Figure S11). Obviously, the anticrossing at the maximum absorbance resonance wavelength (∼625 nm) of CDs in Figure 5e and f indicates that strong coupling occurs between SPPs of silver film (60 nm) and excitons of CDs. The criteria for the strong coupling between plasmons and excitons is 2*g* > |*γ*_e_ + *γ*_pl_|/2, where 2*g*, *γ*_e_, and *γ*_pl_ are the Rabi splitting energy (∼90 meV), the line width of the exciton (∼75 meV), and the line width of the plasmon polariton (∼100 meV for 60 nm thick Ag film).^32−34^ The experimental results shown in Figure 5e were indeed corroborated by theoretical calculations shown in Figure 5g. In addition, the Rabi splitting energy can be increased by increasing the number of excitons; the Rabi splitting energy is proportional to the square root of the number of excitons.^35^ However, we were not able to enlarge the separation between the upper and lower polariton branches in Figure 5e, since the excess amounts of CDs aggregated (leaching out) in the liquid crystal. In other words, only certain number of CDs can be embedded in the liquid crystal. It should be noted that the line width of the surface plasmon polaritons in thin metal films can be tunable by varying the metal film thickness.^35^ In order to show the weak coupling between CDs and surface plasmon polaritons, the thickness of the silver film was decreased to 25 nm (*γ*_pl_ ∼300 meV). In our previous study, we showed that the coupling between excitons and surface plasmon polaritons was affectively tuned by varying the metal film thickness.^35^ A weak coupling (no splitting in the dispersion curve), 2*g* < |*γ*_e_ + *γ*_pl_|/2, was observed between the excitons of CDs and surface plasmon polaritons in Figure 5g and h, where the strong absorption peak of CDs in the liquid crystal was entirely mapped out.

**Figure 6:**
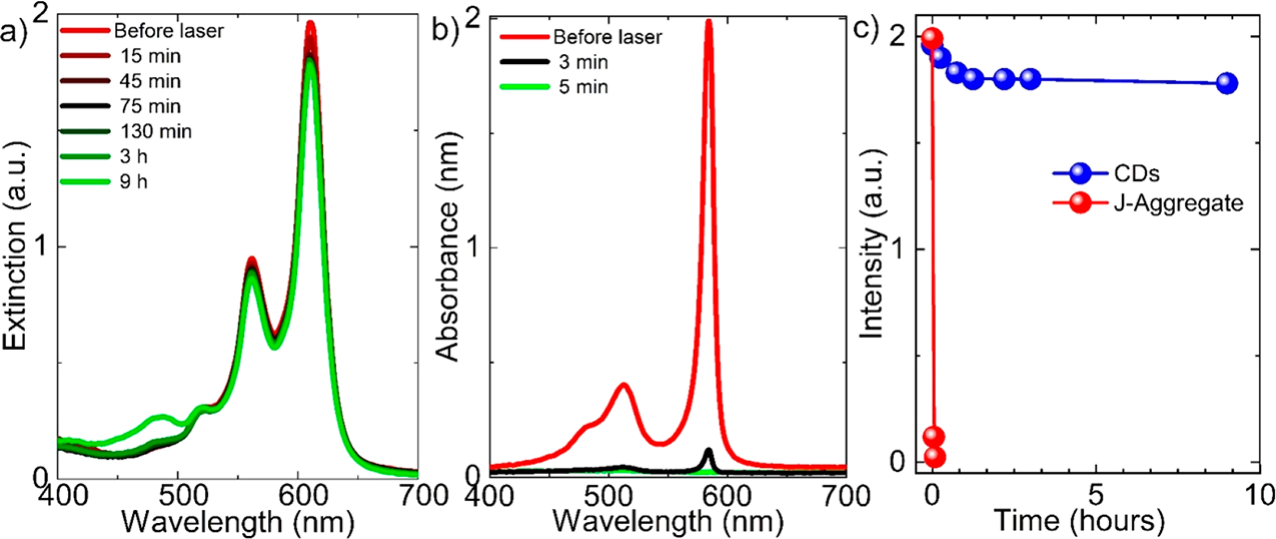
Photostability comparison between CDs and a *J*-aggregate dye, TDBC. (a) Visible spectral change of CDs under the laser light irradiation. CDs are very stable under the laser light irradiation. (b) Visible spectral change of *J*-aggregate dye under the laser light irradiation. (c) The absorbance peak intensity variation of CDs (blue) and *J*-aggregate dye (red) in part a and (b) as a function of time, respectively, during the laser excitation under the same conditions.

Finally, the photostability comparison between CDs and a *J*-aggregate dye, TDBC, a commonly used dye in the strong light–matter interaction studies, was demonstrated in Figure 6. The samples were exposed to a continuous wave laser having a central wavelength of 488 nm and an optical power of 50 mW. After a few minutes of exposure to the laser light, the absorbance peak of the *J*-aggregate dye disappears, which indicates fast photodegradation of the dye under the laser irradiation. Notably, the CDs exhibit outstanding stability at room temperature against the laser exposure. When compared with the commonly used *J*-aggregate dyes in the strong light–matter interaction studies, (i) CDs have better photostability, (ii) CDs and *J*-aggregates have comparable absorption line widths; (iii) CDs can be simply and easily synthesized in high yield from available precursors, but synthesis and isolation of *J*-aggregates suffer from tedious and difficult synthetic procedures; and (iv) theoretical calculations show that CDs and *J*-aggregates with the same oscillator strength on the metal films show very close and comparable Rabi splitting energies. Therefore, we suspect that the colloidal CDs have a bright future in strong light–matter interaction studies.

In summary, we show high-yield hydrothermal synthesis of water-soluble carbon quantum dots with an average diameter of less than 5 nm, high crystallinity, and very narrow line width spectral absorbance and emission, and thus, we demonstrate weak and strong coupling of carbon quantum dots in liquid crystals. The CDs synthesized on a large scale through a facile method show very stable and strong photoluminescence (red emission) with a quantum yield of about 35.4% (in ethanol) and a lifetime of about 2 ns. The excited state dynamics of the synthesized quantum dots have been extensively studied by using ultrafast transient absorption spectroscopy, which explains the excited state dynamics of CDs. Lyotropic liquid crystalline mesophases have been synthesized, and CDs have been dispersed in the liquid crystals. In fact, the CDs have been confined in the hydrophilic domains of the LLC mesophases. The red emitting LLC mesophase–CD film has been placed near a thin metal film. Polarization dependent spectroscopic reflection measurements show that excitons of CDs and surface plasmon polaritons of thin metal film interact strongly and hence the plasmon–exciton hybrid state has been observed in the polariton dispersion curve. When compared with the commonly used *J*-aggregate dyes in the strong light–matter interaction studies, (i) CDs have better photostability; (ii) CDs and *J*-aggregates have comparable absorption line widths; (iii) CDs can be simply and easily synthesized in high yield from available precursors, but synthesis and isolation of *J*-aggregates suffer from tedious and difficult synthetic procedures; and (iv) theoretical calculations show that CDs and *J*-aggregates with the same oscillator strength on the metal films show very close and comparable Rabi splitting energies. Therefore, highly fluorescent carbon quantum dots with very narrow absorption line widths, high quantum yields, high photostability, low toxicity, and tunable emission in the visible and near-infrared region of the electromagnetic spectrum have a bright future in optoelectronic devices, light–matter interaction studies in the nanoscale dimension, nanoscale light sources, bioimaging, biosensing, and therapy.

## Supporting Information Available

Absorbance spectra of CDs, experimental and FDTD simulation details, TEM images, emission maps of CDs, quantum yields of CDs, experimental and theoretical polariton dispersion curves, polarizing optical microscopy images of liquid crystals, and additional references (PDF)
Transparent Peer Review report available (PDF)
