# Supplementary material for: Strong Coupling of Carbon Quantum Dots in Liquid Crystals
Source: J Phys Chem Lett. 2022 Apr 15;13(16):3562–70. doi: 10.1021/acs.jpclett.1c03937 (PMC9059182; doi:10.1021/acs.jpclett.1c03937)
Supplement: Supplementary file 1 — jz1c03937_si_001.pdf [file jz1c03937_si_001.pdf]

Supporting Information for

Strong Coupling of Carbon Quantum Dots in Liquid Crystals

Sema Sarisozen,[a] Nahit Polat,[b] Fadime Mert Balci,[a*] C. Meric Guvenc,[c] Coskun Kocabas,[d,e,f] Halime Gul Yaglioglu,[g*] and Sinan Balci[b*]

[a]Department of Chemistry, Izmir Institute of Technology, Izmir 35430, Turkey

[b]Department of Photonics, Izmir Institute of Technology, Izmir 35430, Turkey

[c]Department of Materials Science and Engineering, Izmir Institute of Technology, Izmir 35430, Turkey

[d]Department of Materials, University of Manchester, Manchester M13 9PL, UK.

[e]National Graphene Institute (NGI), University of Manchester, Manchester M13 9PL, UK

[f]Henry Royce Institute for Advanced Materials, University of Manchester, Manchester M13 9PL, UK

[g]Department of Engineering Physics, Ankara University, Ankara 06100, Turkey

*E-mail: sinanbalci@iyte.edu.tr

*E-mail: fadimemert@iyte.edu.tr

*E-mail: Gul.Yaglioglu@eng.ankara.edu.tr

Table of Contents:

Figure S1. Absorbance spectra of CDs and J-aggregate dye.

Figure S2. Experimental set up used for measuring absorbance of CDs.

Figure S3. Schematic representation of the experimental set up used to generate polariton dispersion curve.

Figure S4. FTIR spectrum of as-synthesized CDs.

Figure S5. pH dependent 2D excitation-emission topographical maps of CDs.

Figure S6. High resolution TEM micrographs of CDs.

Figure S7. Polarizing optical microscopy images of LLC mesophases containing CDs.

Figure S8. Quantum yields (QY) of CDs in water, and ethanol.

Figure S9. Theoretically obtained polariton dispersion curves for CDs, and J-aggregates placed near metal thin films.

Figure S10. UV-vis absorbance spectra of CDs in water.

Figure S11. UV-vis absorbance spectra of CDs in water and in the hexagonal LLC mesophase.

References

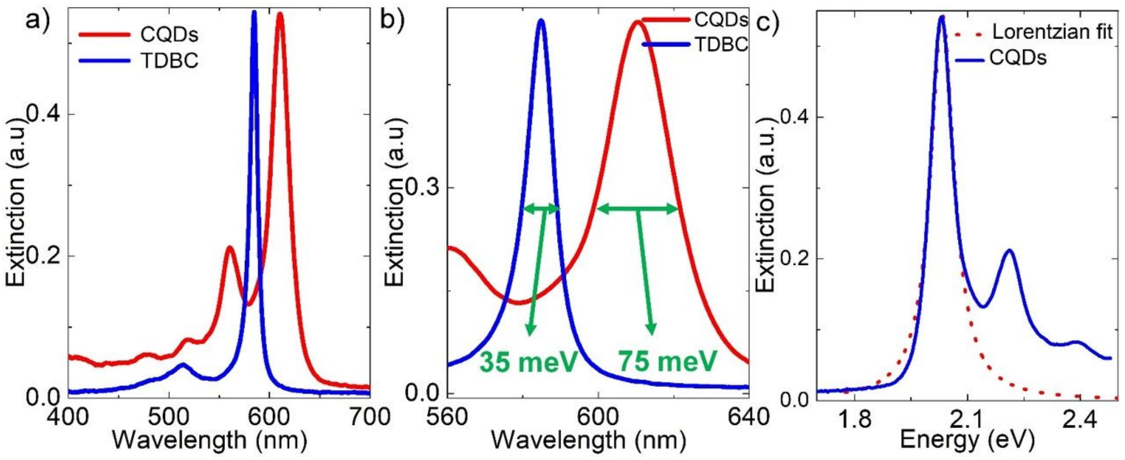

Figure S1. Extinction spectra of CDs and J-aggregate dye, TDBC, used in this study. The full width at half maximum of CDs and J-aggregate dye are 35 meV and 75 meV, respectively. The dotted red line in (c) shows the Lorentzian fit for the CD spectrum.

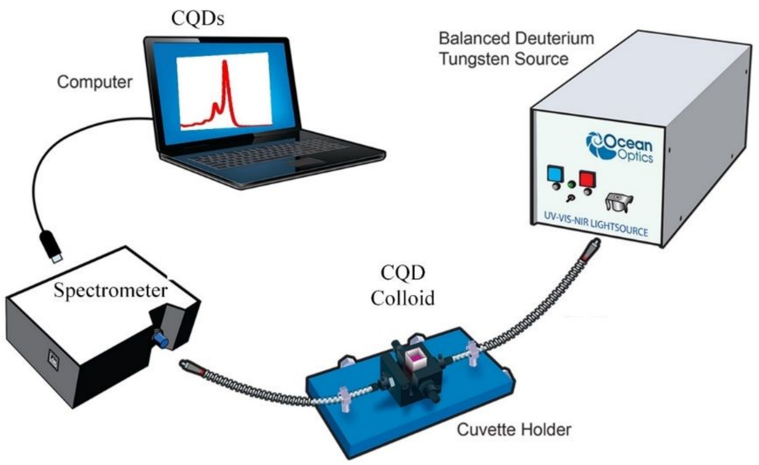

Figure S2. Experimental set up used for measuring absorbance of carbon quantum dots in aqueous medium. In adiditon, the photostabilities of CDs and J-aggregates were studied using the same optical set up. The photostability tests of carbon quantum dots and J-aggregate dyes were performed using a laser having 488 nm wavelength and 50 mW power. Although the CDs were very stable under laser irradiation, after a few minutes exposure to laser light, the absorbance peak of the J-aggregates in the spectra quickly disappeared indicating degradation of the dye under laser irradiation.

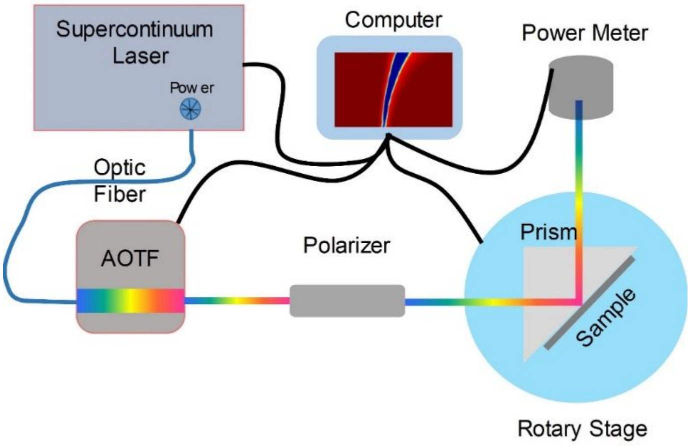

Figure S3. Schematic representation of the experimental set up used to generate polariton dispersion curve in the visible spectrum from CDs in LLC mesophases placed near a thin metal film. In order to study strong coupling between excitons of CDs and surface plasmon polaritons of metal thin film, polarization dependent spectroscopic reflection measurements were taken. A tunable laser light source with a spectral width of around 1 nm; i.e., supercontinuum laser (Koheras-SuperK Versa) with acousto-optic tunable filter working in the visible and near infrared was used as a tunable light source. The rotary stage controls the incident angle with an accuracy less than 0.1^{o}. The incident light is p-polarized to excite surface plasmons on thin metal film.

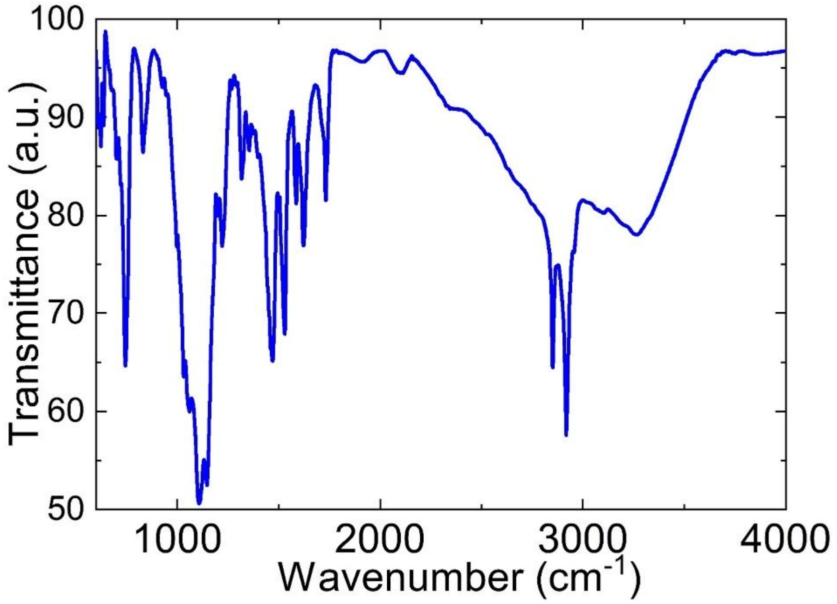

Figure S4. FTIR spectrum of as-synthesized CDs. The FTIR spectrum displays information about the functional groups in CDs. The CDs have indeed the stretching vibration of C-OH (3300 cm^{-1}), the stretching vibration of C-H (2920 cm^{-1}), vibrational absorption of C=O (1730 cm^{-1}), and C=C (1620 cm^{-1}) peaks in the spectrum.[1]

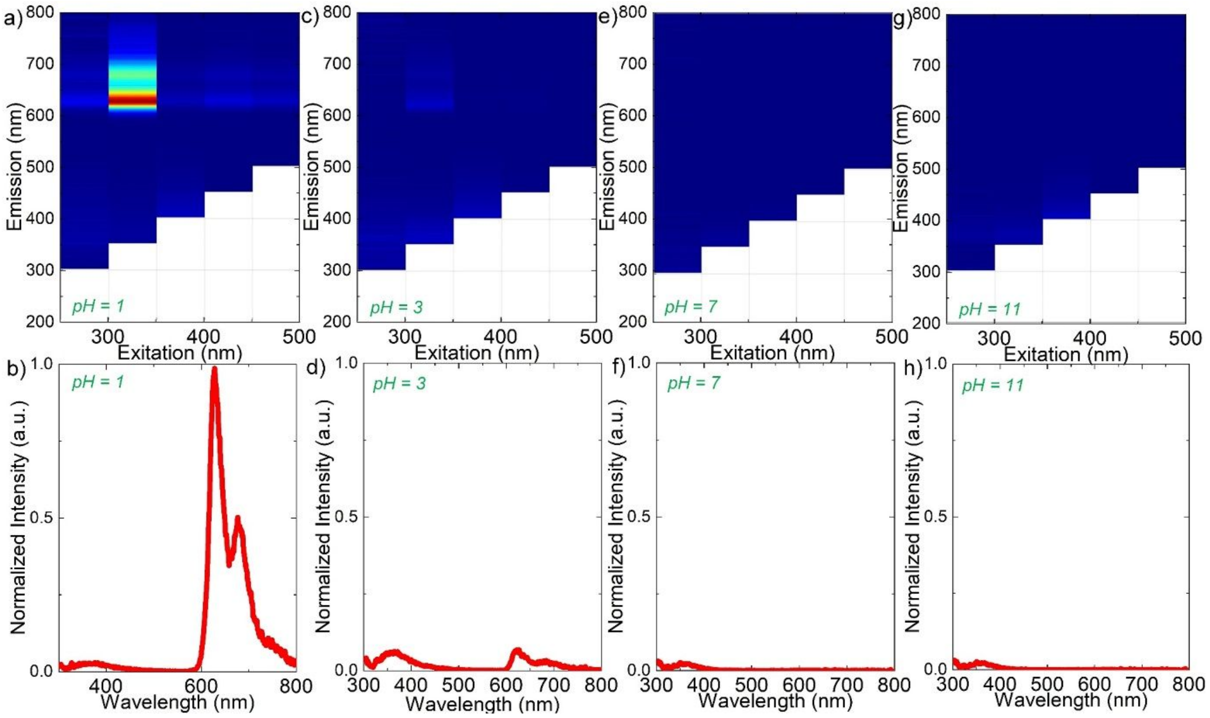

Figure S5. pH dependent 2D excitation-emission topographical maps of CDs. (a-b) pH = 1, (c-d) pH = 3, (e-f) pH = 7, (g-h) pH = 11. The red colored region indicates the maximum PL emission intensity whereas the blue colored region in the map shows the minimum PL emission intensity.

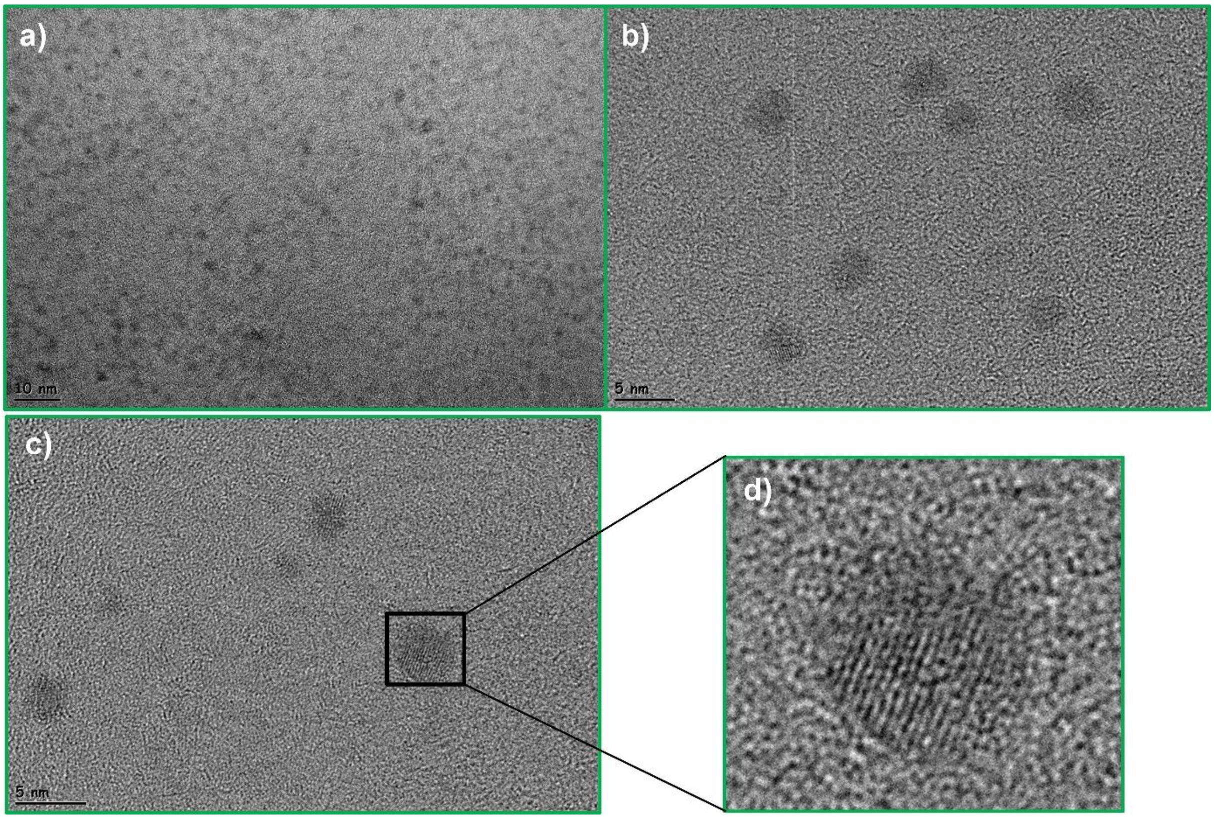

Figure S6. High resolution TEM micrographs of CDs. (a-c) Large area TEM micrographs of CDs showing an average diameter of less than 5 nm. (d) A magnified HRTEM image of a single carbon quantum dot on a carbon coated copper grid.

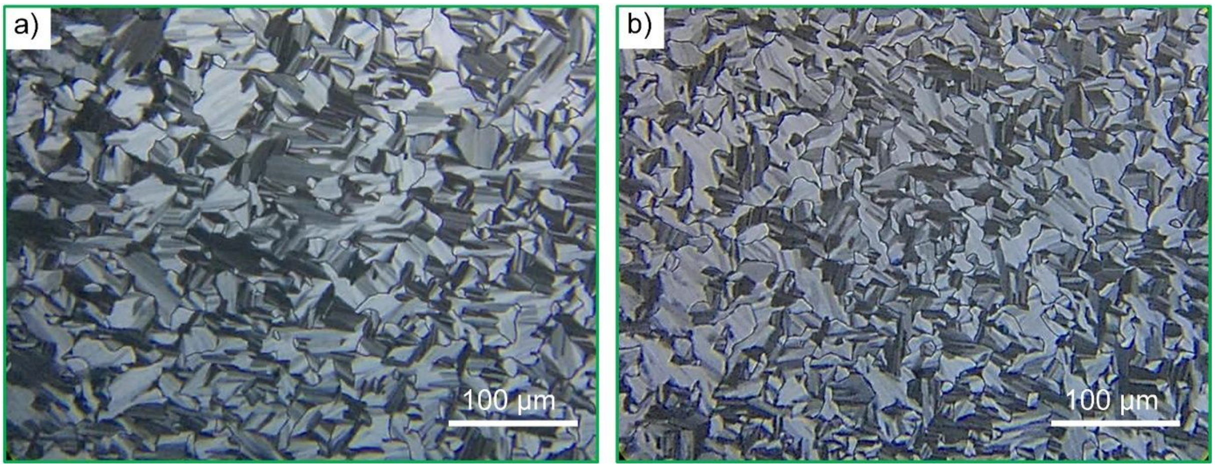

Figure S7. Polarizing optical microscopy images of (a) the hexagonal LLC mesophase, and (b) the hexagonal LLC mesophase containing CDs.

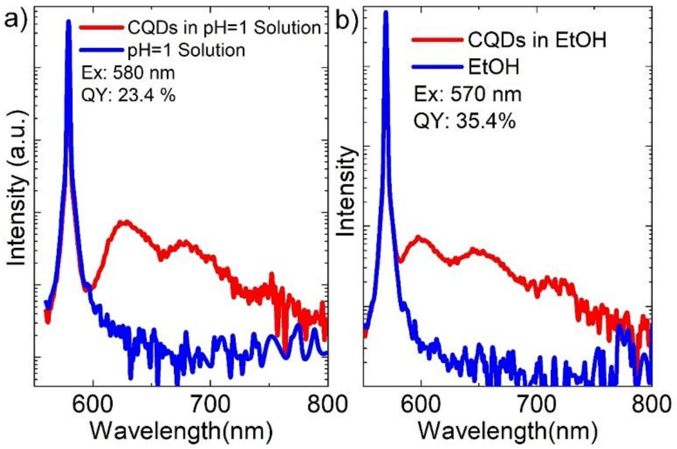

Figure S8. Quantum yields (QYs) of CDs in (a) water, and (b) ethanol. We used time-resolved single photon counting measurement to investigate QY of CDs. The CD colloid in water with a pH of 1, excited with a 580 nm light having a bandwidth of around 3 nm, exhibits strong red fluorescence with a QY of 23.4%. The CD colloid in ethanol shows a red fluorescence with a QY of 35.4%.

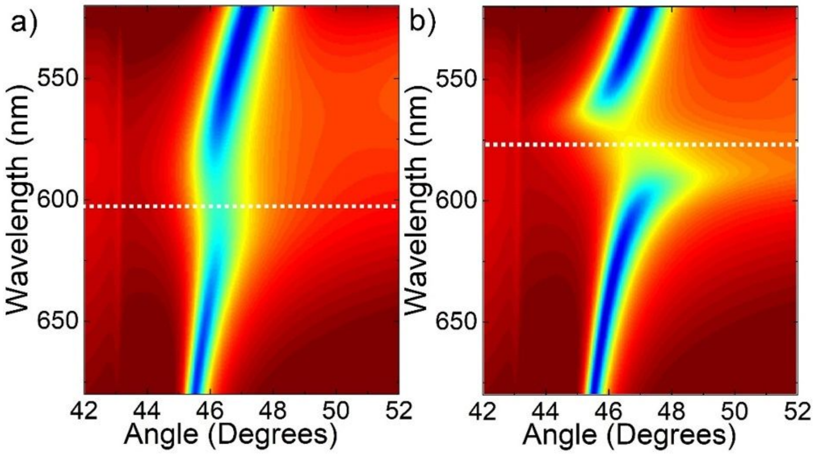

Figure S9. Theoretically obtained polariton dispersion curves for (a) CDs, and (b) J-aggregates placed near metal thin films. The CDs and J-aggregates have the same oscillator strength in FDTD simulations. The red and blue colors in the polariton dispersion curves represent high and low reflectivity, respectively. The Rabi splitting energies obtained from CDs and J-aggregates on thin metal films are comparable.

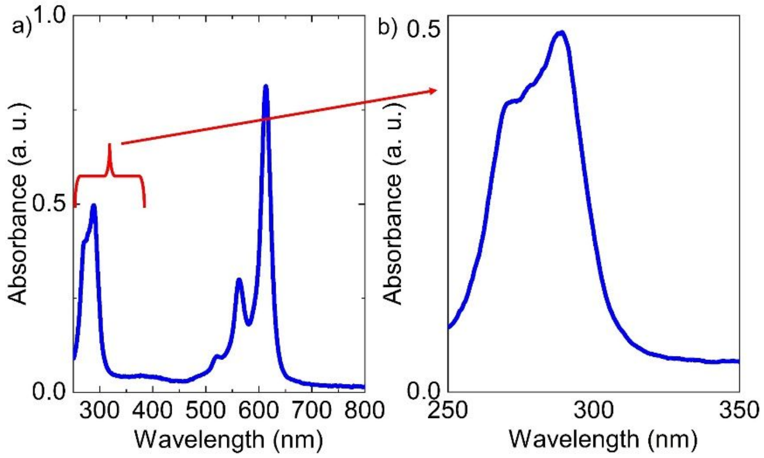

Figure S10. (a) UV-vis absorbance spectra of CDs in water. (b) The ultraviolet region of the spectrum is highlighted. The UV-vis absorption spectra of the CDs were measured by using UV-Vis spectrophotometer. In the UV region, the absorbance bands at around 280 nm are due to the π-π* transitions of C=C, and C=N double bonds. The fluorescence emissions of the CDs in water and ethanol is due to the absorption of the CDs in the lower region of the absorption spectrum (500-650 nm).

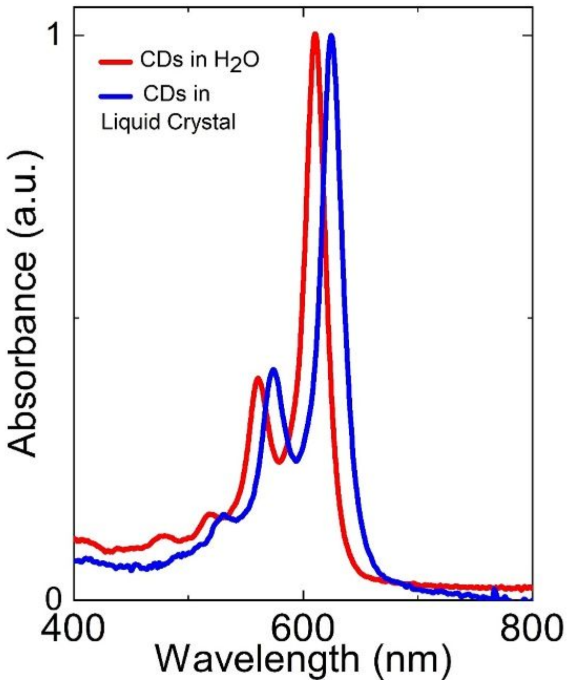

Figure S11. UV-vis absorbance spectra of CDs in water and in the hexagonal LLC mesophase. The wavelengths of the maximum absorbance for the CDs in water and in the hexagonal LLC mesophase are around ~610 nm and ~625 nm, respectivelly.

References

1. Gong, J.; Lu, X.; An, X. Q. Carbon dots as fluorescent off-on nanosensors for ascorbic acid detection. *Rsc Adv* **2015,** *5* (11), 8533-8536.
