# Supplementary material for: Strong Coupling of Carbon Quantum Dots in Liquid Crystals
Source: J Phys Chem Lett. 2022 Apr 15;13(16):3562–70. doi: 10.1021/acs.jpclett.1c03937 (PMC9059182; doi:10.1021/acs.jpclett.1c03937)
Supplement: Supplementary file 2 — jz1c03937_si_002.pdf [file jz1c03937_si_002.pdf]

jz-2021-039379.R1

Name: Peer Review Information for "Strong Coupling of Carbon Quantum Dots in Liquid Crystals"

First Round of Reviewer Comments

Reviewer: 1

Comments to the Author

This is a very interesting experimental work addressing the synthesis of carbon quantum dots with narrow absorption and emission lines and high quantum yield. These carbon quantum dots are further used for strong plasmon-exciton coupling using a 40nm thick Ag film. The paper contains several elements of novelty, is well-written, timely, and interesting. I, therefore, recommend it for publication after fixing several minor issues:

1) It is not clear why the authors necessarily need LLC together with CDs to obtain strong coupling. Since it was not discussed, I encourage the authors to explain the motivation behind using LLC more clearly. Can strong coupling be reached without LLC?

2) In relation to pH-dependence of CDs absorption/emission, would strong coupling also be pH-dependent? Is that the reason for using LLC with a high H2SO4 content?

3) Finally, there are several typos in the manuscript:

- page 5, line 26, "... conlude ..."

- page 9, line 41, "...be be..."

- It also seems the "RESULTS SECTION" in page 10 is missing. Part of this section is just a repetition of the EXPERIMENTAL SECTION. This overlap could be minimized in my opinion, for better readability.

Reviewer: 2

Comments to the Author

The paper entitled „Strong Coupling of Carbon Quantum Dots in Liquid Crystal" studies linear optical spectra and transient absorption of carbon dots and of carbon dots deposited on a thin silver film.

One of the central claims of the paper (see abstract) is that it "demonstrate(s) strong coupling of carbon quantum dots and surface plasmon polaritons in lyotropic crystalline mesophases".

Among the characteristics of such strong couplings are (a) the emergence of new, hybrized modes in the optical spectra showing up as distinct level splittings (anticrossings) as schematically depicted in Fig.5b or (b) the emergence of coherent energy exchange phenomena between the coupled modes. While

level splittings have been studied extensively in a wide variety of systems, coherent energy exchange ("Rabi oscillations in time domain") have been seen for very few selected systems only.

In the paper the auhors the discuss the synthesis of carbon dots in liquid crystal mesophases (LLCs). They present TEM images of selected carbon dots (Fig. 2) and study linear excitation and emission spectra of carbon dots in water and in LLCs. The spectra seem to reasonably match the expectations from a displaced harmonic oscillator model that usually describes the coupling of excitons to a dominant vibrational mode with large Huang-Rhys factor. This is a well established model for such dots. Transient absorption spectra (Fig. 4) show mainly a long-lived (1.5 ns) ground state bleaching, in addition to some rather weak sub-ps component that may be related to energy relaxation in the dots.

Angle-resolved linear light scattering spectra of carbon dots deposited on a silver film are studied in Kretschmann configuration (Fig. 5e). These spectra show slight (it is not possible to say what "slight" means exactly since the color scale bar of the image is only given in relative units) reduction in light scattering intensity at the crossing angle between the SPP and the exciton dispersion relation. Even without quantitative information about the light scattering intensity, it is evident that the data in Fig.5e do NOT show level splittings or anticrossings. As such, these data do certainly not provide evidence for strong coupling between carbon dot excitons and SPP. This is actually confirmed by the simulations (which do not match the experimental data too well…) which show no sign of a strong coupling.

This implies that the data in the paper do not support the claim (strong coupling…) that is made in the abstract. I therefore do not recommend this manuscript for publication in its present form.

Some more technical comments:

It is certainly correct to say (p. 10) that the Kretschmann configuration is often used to excite SPPs. Yet, this is usually not related to a "strong coupling". In contrast the coupling between the propagating incident wave and the plasmon mode is usually "weak", implying that is well described within first order perturbation theory.

The sentence on p.9 "…the plane wave moves in z-direction" was not clear to me.

The next sentence: "SPPs… very excited by varying the incidence angle…" needs more detailed explanation.

Author's Response to Peer Review Comments:

Referee 1

Comments:

This is a very interesting experimental work addressing the synthesis of carbon quantum dots with narrow absorption and emission lines and high quantum yield. These carbon quantum dots are further used for strong plasmon-exciton coupling using a 40nm thick Ag film. The paper contains several elements of novelty, is well-written, timely, and interesting. I, therefore, recommend it for publication after fixing several minor issues:

Our response: We would like to thank the referee for the comments about our manuscript.

Comments:

1) It is not clear why the authors necessarily need LLC together with CDs to obtain strong coupling. Since it was not discussed, I encourage the authors to explain the motivation behind using LLC more clearly. Can strong coupling be reached without LLC?

Our response: In this work, we hydrothermally synthesize carbon quantum dots from ortho-phenylenediamine (OPD) and L-phenylalanine (LPA) in water-sulphuric acid mixture at a high temperature, 210 ℃. The carbon quantum dots are indeed in colloidal form and they are soluble in water. In addition, we synthesize lyotropic liquid crystalline (LLC) mesophases from sulphuric acid, surfactant, C12E10, and ethanol. The synthesized CDs and the synthesized liquid crystals are both in acidic medium. Therefore, we are able to disperse carbon quantum dots in LLC mesophases. We also try to prepare CDs in polymer films, for example polyvinyl alcohol (PVA), but we were not able to prepare carbon quantum dot thin films, which is due to the degredation of the polymer film in the acidic medium of the CDs. We are able to control dispersion of CDs in liquid crystalline lattice in three dimension. The CDs can be confined in the hydrophilic domains of the liquid crystals. This can not be achieved by using other dispersing materials for example polymer thin films.

Comments:

2) In relation to pH-dependence of CDs absorption/emission, would strong coupling also be pH-dependent? Is that the reason for using LLC with a high H2SO4 content?

Our response: Yes, strong coupling is pH dependent. The CDs have very sharp absorption in acidic medium. Figure S5 gives information about the pH dependent optical properties of the carbon quantum dots. In the acidic medium, the CDs strongly emit red light. Yes, the main reason for using LLC with a high H2SO4 content is that the carbon quantum dots emit red ligth with a high quantum yield in the acidic medium and hence we were able to study coupling between excitons of CDs and plasmons of thin metal film.

Comments:

3) Finally, there are several typos in the manuscript:

- page 5, line 26, "... conlude ..."

Our response: We corrected ‘conlude’ as ‘conclude’

- page 9, line 41, "...be be..."

Our response: We removed one of the ‘be’ from the text.

- It also seems the "RESULTS SECTION" in page 10 is missing. Part of this section is just a repetition of the EXPERIMENTAL SECTION. This overlap could be minimized in my opinion, for better readability.

Our response: We now seperated experimental section from the result section by adding (RESULTS AND DISCUSSION).

Referee 2

Comments:

The paper entitled „Strong Coupling of Carbon Quantum Dots in Liquid Crystal" studies linear optical spectra and transient absorption of carbon dots and of carbon dots deposited on a thin silver film. One of the central claims of the paper (see abstract) is that it "demonstrate(s) strong coupling of carbon quantum dots and surface plasmon polaritons in lyotropic crystalline mesophases". Among the characteristics of such strong couplings are (a) the emergence of new, hybrized modes in the optical spectra showing up as distinct level splittings (anticrossings) as schematically depicted in Fig.5b or (b) the emergence of coherent energy exchange phenomena between the coupled modes. While level splittings have been studied extensively in a wide variety of systems, coherent energy exchange ("Rabi oscillations in time domain") have been seen for very few selected systems only.

In the paper the auhors the discuss the synthesis of carbon dots in liquid crystal mesophases (LLCs). They present TEM images of selected carbon dots (Fig. 2) and study linear excitation and emission spectra of carbon dots in water and in LLCs. The spectra seem to reasonably match the expectations from a displaced harmonic oscillator model that usually describes the coupling of excitons to a dominant vibrational mode with large Huang-Rhys factor. This is a well established model for such dots. Transient absorption spectra (Fig. 4) show mainly a long-lived (1.5 ns) ground state bleaching, in addition to some rather weak sub-ps component that may be related to energy relaxation in the dots.

Angle-resolved linear light scattering spectra of carbon dots deposited on a silver film are studied in Kretschmann configuration (Fig. 5e). These spectra show slight (it is not possible to say what "slight" means exactly since the color scale bar of the image is only given in relative units) reduction in light scattering intensity at the crossing angle between the SPP and the exciton dispersion relation. Even without quantitative information about the light scattering intensity, it is evident that the data in Fig.5e do NOT show level splittings or anticrossings. As such, these data do certainly not provide evidence for strong coupling between carbon dot excitons and SPP. This is actually confirmed by the simulations (which do not match the experimental data too well…) which show no sign of a strong coupling.

This implies that the data in the paper do not support the claim (strong coupling…) that is made in the abstract. I therefore do not recommend this manuscript for publication in its present form.

Our response: We would like to thank the referee for the comments about our manuscript. In this work, we seperately synthesize carbon quantum dots and liquid crystals and then disperse carbon quantum dots in the hydrophilic domains of the liquid

crystals. Therefore, we can control organization of carbon quantum dots in the liquid crystal mesophases. In this work, we place carbon quantum dots dipersed in the liquid crystals near a metal film and study interaction of excitons of carbon quantum dots and surface plasmon polaritons on thin metal films.

In the previous version of the manuscript in Figure 5e, the experimental data show strong coupling at round 625 nm, however, the simulation shown in Figure 5f doesnt match the experimental data (the splitting is at around 610 nm). Therefore, we agree with the reviewer on this point and we tried to find the main reason why the splitting in the experimental result doesnt match the splitting in the simulation.

In water, the maximum absorption of the carbon quantum dots is around 610 nm, see Figure 3a. Since we study the strong coupling in the liquid crystalline mesophases, we now have measured the absoption of the carbon quantum dots in the liquid crystal mesophases and we provide now this data in Figure S11. In fact, the maximum absorption of the carbon quantum dots in liquid crystalline phases is at around 625 nm. Therefore, we resimulated the experimental results shown in Figure 5e by taking the maximum absorption of the carbon quantum dot as 625 nm. In the previous version of the manuscript, the simulation was performed by taking the absorbance of CDs in water (610 nm). Now, the experimental polariton dispersion curve in Figure 5e and the new simulation shown in Figure 5f support each other.

Comments:
Some more technical comments:
It is certainly correct to say (p. 10) that the Kretschmann configuration is often used to excite SPPs. Yet, this is usually not related to a “strong coupling”. In contrast the coupling between the propagating incident wave and the plasmon mode is usually “weak”, implying that is well described within first order perturbation theory.
Our response: We agree with the referee and we now removed ‘strong coupling regime’ in the excitation of SPPs in page 19. This is also making confusion with the strong coupling studied in this work between the excitons of the carbon quantum dots and surface plasmon polaritons of the metal thin films.

Comments:
The sentence on p.9 “…the plane wave moves in z-direction” was not clear to me.
Our response: We agree with the referee and we now modify this sentence as ‘In the simulations, a plane wave was used.’

Comments:
The next sentence: “SPPs… very excited by varying the incidence angle…” needs more detailed explanation.
Our response: We agree with the referee and we now explain this point in detail. We added the following further explanation: ‘For example, when the incidence angle is 45° for a bare silver film, the SPP resonance wavelength is around 600 nm.’

Changes to the manuscript:

1-) The length of the abstract has been shortened.

2-) Page 9, 'For example, when the incidence angle is 45° for a bare silver film, the SPP resonance wavelength is around 600 nm.' has been added.

3-) Page 19, 'It should be also noted here that CDs in water and in LLC mesophases have the maximum absorbance resonance wavelengths of around 610 nm and 625 nm, respectively (see Supporting Information, Figure S11).' has been added.

4-) After obtaining maximum absorbance of carbon quantum dots in liquid crystal as 625 nm, Figure 5f has been redrawn.

5-) Figure S11 has been added to the Supporting Information:

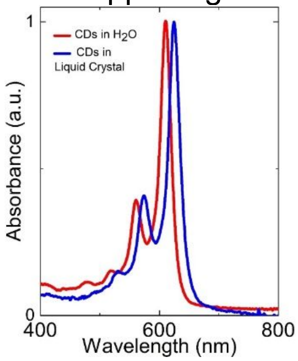

Figure S11. UV-vis absorbance spectra of CDs in water and in the hexagonal LLC mesophase. The wavelengths of the maximum absorbance for the CDs in water and in the hexagonal LLC mesophase are around ~610 nm and ~625 nm, respectivelly.

jz-2021-039379.R2

Name: Peer Review Information for "Strong Coupling of Carbon Quantum Dots in Liquid Crystals"

Second Round of Reviewer Comments

Reviewer: 2

Comments to the Author

The authors have responded to the reviewer comments.

I very much appreciate the achievements of the authors in the synthesis of carbon quantum dots.

I think that my first report has not yet clearly expressed the problem that I have with this manuscript in its present form.

The authors claim, in the title of their paper, "Strong coupling of carbon quantum dots...". I understand that the authors claim that they "demonstrate strong coupling of carbon quantum dots (CQDs)" and plasmon polaritons in liquid crystalline mesophases.

I also understand that this claim is based on a slight difference in angle-resolved reflectivity spectra of CQDs on a thin silver film (Fig. 5e) and angle-resolved reflectivity spectra of the bare silver film (Fig. 5c).

The term "strong coupling" is clearly defined in the quantum optics literature. Two systems are said to be "strongly coupled" if the coupling strength g exceeds the losses of the two systems.

In this strong coupling regime, the coupling results in two spectrally well resolved peaks in, for instance, the spectrum of the light that is reflected from the surface.

For a formal definition of strong coupling see - for instance - C. Carlsson et al., Physical Review B 104, 125424 (2021) and references therein.

The characteristing peak splitting is *not* seen in the spectra shown in Fig. 5e. Therefore the system studied in the present manuscript is *not* in the strong coupling regime.

Therefore, the claims made by the authors in the title, in the abstract and in the main paper about "strong coupling" are not substantiated by the experimental data.

I therefore do not recommend to publish this manuscript in its present form.

Author's Response to Peer Review Comments:

Editorial Revision:

Please also make these non-scientific changes:

1.) Supporting Information Statement: A brief, nonsentence description of the actual contents of each supporting information file is required. This description should be labeled Supporting Information and should appear before the Acknowledgement and Reference sections. Examples of sufficient and insufficient descriptions are as follows:

*Examples of sufficient descriptions: “Supporting Information: 1H NMR spectra for all compounds” or “Additional experimental details, materials, and methods, including photographs of experimental setup”.

*Examples of insufficient descriptions: “Supporting Information: Figures S1-S3” or “Additional figures as mentioned in the text”.

Our response: We now have added nonsentence description of the actual contents of each supporting information file.

2.) Headers: Remove the section heading(s) throughout the manuscript (you can leave headings for your TOC graphic, Abstract, and Methods).

Our response: We now have removed the section headings throughout the manuscript.

3.) Please include postal codes/country in the author affiliations in the manuscript file.

Our response: We now have included postal codes/country in the author affiliations in the manuscipt file.

Referee 2

Comments:

The authors have responded to the reviewer comments. I very much appreciate the achievements of the authors in the synthesis of carbon quantum dots. I think that my first report has not yet clearly expressed the problem that I have with this manuscript in its present form. The authors claim, in the title of their paper, "Strong coupling of carbon quantum dots...". I understand that the authors claim that they "demonstrate strong coupling of carbon quantum dots (CQDs)" and plasmon polaritons in liquid crystalline mesophases. I also understand that this claim is based on a slight difference in angle-resolved reflectivity spectra of CQDs on a thin silver film (Fig. 5e) and angle-resolved reflectivity spectra of the bare silver film (Fig. 5c). The term "strong coupling" is clearly defined in the quantum optics literature. Two systems are said to be "strongly coupled" if the coupling strength g exceeds the losses of the two systems. In this strong coupling regime, the coupling results in two spectrally well resolved peaks in, for instance, the spectrum of the light that is reflected from the surface. For a formal definition of strong coupling see - for instance - C. Carlsson et al., Physical Review B 104, 125424 (2021) and references therein. The characteristing peak splitting is *not* seen in the spectra shown in Fig. 5e. Therefore the system studied in the present manuscript is *not* in the strong coupling regime. Therefore, the claims made by the authors in the title, in the abstract and in the main paper about "strong coupling" are not substantiated by the experimental data. I therefore do not recommend to publish this manuscript in its present form.

Our response: We would like to thank the referee for the comments about our manuscript. We have now performed additional experiments and observed transition from weak coupling to strong coupling by varying the silver metal film thickness. In a similar way, in our previous study, we tuned plasmon exciton coupling from weak coupling to strong coupling by varying the metal film thickness. We have now added new figures in Figure 5 showing weak and strong coupling of CDs with the surface plasmon polaritons on the silver film. When the silver film thickness is 25 nm, a weak coupling was observed. In this case, the absorption bands of the CDs were entirely mapped out in the dispersion curve and no splitting of the energy levels were observed. This is weak coupling between the excitons of CDs and surface plasmon polaritons of the silver film. When the silver film thickness is 60 nm, a transparency deep in the dispersion curve at around the resonance wavelength of CDs were observed. This is strong coupling between the excitons of the CDs and the surface plasmon polaritons of the silver film. The experimental results were corroborated by theoretical calculations. We believe that we clearly show both weak and strong coupling of CDs in the liquid crystal placed near a metal thin film. We added the following text to the manuscript:

Obviously, the anticrossing at the maximum absorbance resonance wavelength (~625 nm) of CDs in Figures 5e and 5f indicate that strong coupling occurs between SPPs of silver film (60 nm) and excitons of CDs. The criteria for the strong coupling between plasmons and excitons is $2g > |\gamma_e + \gamma_{pl}|$ where $2g$, γ_e, and γ_{pl} are the Rabi splitting energy (~90 meV), the linewidth of the exciton (~75 meV), and the linewidth of the plasmon polariton (~100 meV for 60 nm thick Ag film).[1-2] The experimental results shown in Figure 5e were indeed corroborated by theoretical calculations shown in Figure 5g. In addition, the Rabi splitting energy can be increased by increasing the number of excitons; Rabi splitting energy is proportional to the square root of the number of excitons.[3] However, we were not able to enlarge the separation between the upper and lower polariton branches in Figure 5e since the excess amounts of CDs aggregated (leaching out) in the liquid crystal. In other words, only certain number of CDs can be embedded in the liquid crystal. It should be noted that the linewidth of the surface plasmon polaritons in thin metal films can be tunable by varying the metal film thickness.[3] In order to show the weak coupling between CDs and surface plasmon polaritons, the thickness of the silver film was decreased to 25 nm (γ_{pl} ~300 meV). In our previous study, we showed that the coupling between excitons and surface plasmon polaritons was affectively tuned by varying the metal film thickness.[3] A weak coupling (no splitting in the dispersion curve), $2g < |\gamma_e + \gamma_{pl}|$, was observed between the excitons of CDs and surface plasmon polaritons in Figures 5g and 5h where the strong absorption peak of CDs in the liquid crystal was entirely mapped out.

List of Changes:

1-) New figures (Figure 5g and 5h) were added. In addition, figure captions were added.

2-) In the results and discussion section of the manuscript, the new figures were discussed and explained in detail. The following text was added.

Obviously, the anticrossing at the maximum absorbance resonance wavelength (~625 nm) of CDs in Figures 5e and 5f indicate that strong coupling occurs between SPPs of silver film (60 nm) and excitons of CDs. The criteria for the strong coupling between plasmons and excitons is $2g > |\gamma_e + \gamma_{pl}|$ where $2g$, γ_e, and γ_{pl} are the Rabi splitting energy (~90 meV), the linewidth of the exciton (~75 meV), and the linewidth of the plasmon polariton (~100 meV for 60 nm thick Ag film).[1-2] The experimental results shown in Figure 5e were indeed corroborated by theoretical calculations shown in Figure 5g. In addition, the Rabi splitting energy can be increased by increasing the number of excitons; Rabi splitting energy is proportional to the square root of the number of excitons.[3] However, we were not able to enlarge the separation between the upper and lower polariton branches in Figure 5e since the excess amounts of CDs aggregated (leaching out) in the liquid crystal. It should be noted that the linewidth of the surface plasmon polaritons in thin metal films can be tunable by varying the metal film thickness.[3] In order to show the weak coupling between CDs and surface plasmon polaritons, the thickness of the silver film was decreased to 25 nm (γ_{pl} ~300 meV). In our previous study, we showed that the coupling between excitons and surface plasmon polaritons was affectively tuned by varying the metal film thickness.[3] A weak coupling (no splitting in the dispersion curve), $2g < |\gamma_e + \gamma_{pl}|$, was observed between the excitons of CDs and surface plasmon polaritons in Figures 5g and 5h where the strong absorption peak of CDs in the liquid crystal was entirely mapped out.

3-) The following new references were added.

1. Zengin, G.; Wersall, M.; Nilsson, S.; Antosiewicz, T. J.; Kall, M.; Shegai, T. Realizing Strong Light-Matter Interactions between Single-Nanoparticle Plasmons and Molecular Excitons at Ambient Conditions. *Phys Rev Lett* 2015, *114* (15).
2. Carlson, C.; Salzwedel, R.; Selig, M.; Knorr, A.; Hughes, S. Strong coupling regime and hybrid quasinormal modes from a single plasmonic resonator coupled to a transition metal dichalcogenide monolayer. *Phys Rev B* 2021, *104* (12).
3. Balci, S.; Kocabas, C.; Ates, S.; Karademir, E.; Salihoglu, O.; Aydinli, A. Tuning surface plasmon-exciton coupling via thickness dependent plasmon damping. *Phys Rev B* 2012, *86* (23), 235402.

jz-2021-039379.R3

Name: Peer Review Information for "Strong Coupling of Carbon Quantum Dots in Liquid Crystals"

Third Round of Reviewer Comments

Reviewer: 2

Comments to the Author

I appreciate that the authors present new measurements and now give quantitative criteria for what they mean by “strong coupling”.

I nevertheless maintain my claim that the measurements do not show strong coupling. To make my point clear, I add the following illustrative figure from the Review by Barnes and Törmä. The **simulations** reported in Fig. 5 are representative of an intermediate coupling regime (see subfigure (c) below. The coupling that is shown in the **experimental** data in Fig. 5 is even less pronounced.

I quote the caption from the figure shown below. “(c)–(d) Distortions of the dispersion when approaching strong coupling, but splitting not yet being visible like in (c), may lead to slight back-bending see (d) when the results are shown as an angle-plot with angle-scan used for plotting minima.”

Rep. Prog. Phys. **78** (2015) 013901 Report on Progress

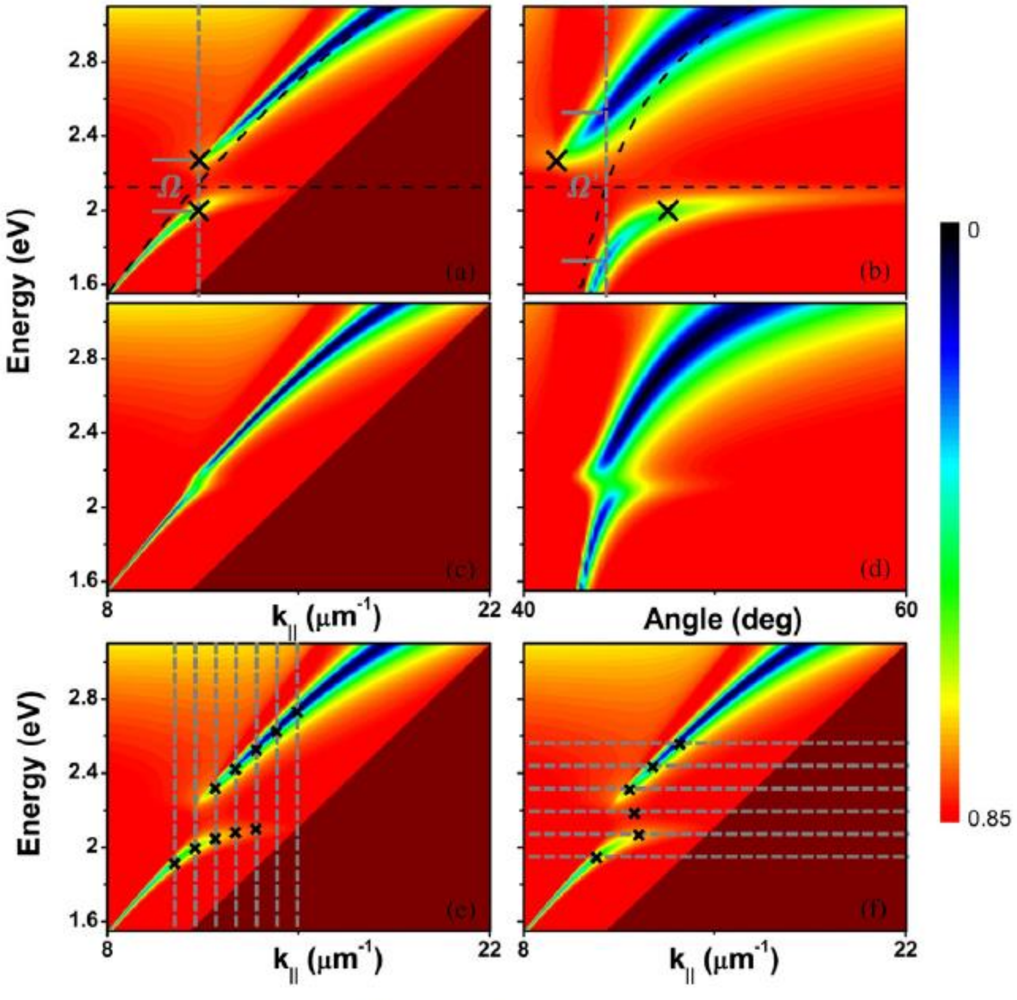

Figure 11. (*a*)–(*b*) Reflectance plotted as a function of: left column, frequency (energy) versus in-plane wavevector; and right column, frequency (energy) versus incident angle. Due to the mapping $k = (2\pi/\lambda)\, n_p \sin\theta$ in reflectometry experiments, points corresponding to one k-vector but different frequencies become shifted with respect to each other in an angle plot. The Rabi splitting/normal mode splitting Ω is determined as the difference of the two branches at the resonance point, see (*a*). Attempting to determine the splitting Ω' from the angle-plot will lead to an overestimation of the splitting, see (*b*). (*e*)–(*f*) The vertical/horisontal lines depict wavelength (frequency)/angle scans, respectively: the minima determined from these scans differ as shown by the black crosses in (*e*)–(*f*). Finite linewidth of the normal modes is required for this difference to arise. (*c*)–(*d*) Distortions of the dispersion when approaching strong coupling, but splitting not yet being visible like in (*c*), may lead to slight back-bending see (*d*) when the results are shown as an angle-plot with angle-scan used for plotting minima.

In addition, the claim of “strong coupling” that is made in the paper is inconsistent with the criterion and the numbers that are now quoted in the paper:

a) Strong coupling condition: $2g > \gamma_e + \gamma_{pl}$

b) Numbers: $2g = 90$ meV, $\gamma_e = 75$ meV, $\gamma_{pl} = 100$ meV

c) Result: $90 \text{ meV} < 175 \text{ meV}$

d) Conclusion: The system is not in the strong coupling regime (even if I accept the numbers that are given by the authors).

The claim of strong coupling should be withdrawn.

Author's Response to Peer Review Comments:

Referee 2

Comments:

I appreciate that the authors present new measurements and now give quantitative criteria for what they mean by “strong coupling”.

a) Strong coupling condition: 2g > γe + γpl

b) Numbers: 2g = 90 = meV, γe = 75 meV, γpl = 100 meV,

c) Result: 90 meV < 175 meV

d) Conclusion: The system is not in the strong coupling regime (even if I accept the numbers that are given by the authors).

The claim of strong coupling should be withdrawn.

Our response: We would like to thank the referee for the comments about our manuscript. The strong coupling condition as stated in *Nature Physics 2006, 2 (2), 81-90 by Khitrova et al.* (Page 83, Figure 2)[1] is that ‘The commonly used condition for strong coupling, 2g ≥ (κ+γdot)/2’. In the previous version of our manuscript, the strong coupling condition was stated as $2g > |\gamma_e + \gamma_{pl}|$. The formula should be as $2g > |\gamma_e + \gamma_{pl}|/2$ as stated in *Nat Phys 2006, 2 (2), 81-90 by Khitrova et al.* In the previous version of the manuscript, in the formula, ½ was missing. We corrected this in the new version of the manuscript. Now, 90 meV is comparable with 175/2 meV and hence the system is in the strong coupling regime.

List of Changes:

1-) The following new reference was added.

Khitrova, G.; Gibbs, H. M.; Kira, M.; Koch, S. W.; Scherer, A. Vacuum Rabi splitting in semiconductors. *Nat Phys* **2006,** *2* (2), 81-90.

2-) The $2g > |\gamma_e + \gamma_{pl}|$ is replaced with $2g > |\gamma_e + \gamma_{pl}|/2$.
